# 7-deacetylgedunin suppresses inflammatory responses through activation of Keap1/Nrf2/HO-1 signaling

**DOI:** 10.18632/oncotarget.19017

**Published:** 2017-07-05

**Authors:** Jian-Yu Chen, Guo-Yuan Zhu, Xiao-Hui Su, Rui Wang, Juan Liu, Kangsheng Liao, Rutong Ren, Ting Li, Liang Liu

**Affiliations:** ^1^ State Key Laboratory of Quality Research in Chinese Medicine, Macau Institute for Applied Research in Medicine and Health, Macau University of Science and Technology, Macau, China

**Keywords:** 7-deacetylgedunin, anti-inflammation, Keap1, Nrf2, HO-1

## Abstract

Macrophages play a critical role in a variety of inflammatory diseases. Activation of Keap1/Nrf2/HO-1 signaling results in inactivation of macrophages and amelioration of inflammatory and autoimmune conditions. Hence, discovery for the activators of Keap1/Nrf2/HO-1 signaling has become a promising strategy for treatment inflammatory diseases. In the current study, the anti-inflammatory potential of 7-deacetylgedunin (7-DGD), a limonin chemical isolated from the fruits of *Toona sinensis* (A. Juss.) Roem, was intensively examined *in vivo* and *in vitro* for the first time. Results showed that 7-DGD alleviated mice mortality induced by LPS. Mechanistic study showed that 7-DGD suppressed macrophage proliferation *via* induction of cell arrest at the G0/G1 phase. Furthermore, 7-DGD inhibited iNOS expression, which is correlated with the increases of NQO1, HO-1 and UGT1A1 mRNA expression as well as HO-1 protein expression level in the cells. More importantly, 7-DGD markedly decreased Keap1 expression, promoted p62 expression, and facilitated Nrf2 translocation and localization in the nucleus of macrophages, and in turn up-regulates these anti-oxidant enzymes expression, eventually mediated anti-inflammatory effect. Collectively, 7-DGD suppresses inflammation *in vivo* and *in vitro*, indicating that the compound is valuable for further investigation as an anti-inflammatory agent in future.

## INTRODUCTION

Inflammation is one of the major pathogenic events in a variety of diseases in which macrophages play crucial roles [1]. When macrophages are stimulated by inflammatory stimuli, they become activated and secrete pro-inflammatory mediators, growth factors, bioactive lipids, hydrolytic enzymes, reactive oxygen intermediates, and nitric oxide [2] and promote the synthesis of prostaglandins [3]. Consequently, inflammatory responses and even disease conditions are induced. Moreover, inducible NOS (iNOS) and cyclooxygenase-2 (COX-2) are two key factors in the processes of inflammation and disease progression; in the quiescent stage of macrophages, both remain unexpressed. In contrast, when quiescent cells are stimulated by interferon gamma (IFN-γ), lipopolysaccharides (LPS) and other pro-inflammatory cytokines [4], iNOS and COX-2 are overexpressed, and the increased expression of iNOS and COX-2 causes the release of NO and PGE_2_, inducing inflammatory reactions [5].

Oxidative stress plays important roles in both inflammatory and anti-inflammatory responses by activating various transcription factors such as NF-κB, AP-1, p53, HIF-1α, PPAR-γ, β-catenin/Wnt, and NF-E2-related factor 2 (Nrf2), which regulate more than 500 different genes of growth factors, inflammatory cytokines, chemokines, cell cycle regulatory molecules, and anti-inflammatory molecules [6]. Heme oxygenase-1(HO-1), a stress-inducible protein in the Keap1/Nrf2/HO-1 pathway, is induced by various oxidative and inflammatory signals, subsequently inducing anti-inflammatory activity. Therefore, HO-1 functions as an adaptive cellular response against inflammation and oxidative injury and it is regulated by Nrf2 [7]. Under nonstressed conditions, Nrf2 is constantly ubiquitinated by the Cul3-Keap1 ubiquitin E3 ligase complex and rapidly degraded [7]. When the cell experiences conditions of stress or in the presence of electrophiles, the cysteine residues in Keap1 are modified, resulting in Nrf2 release. Free Nrf2 accumulates in the cytosol, and then translocates to the nucleus where it binds to the ARE [8], activating the transcription of chemopreventive genes, including UDP-glucuronosyltransferases (UGTs), detoxifying enzymes NAD(P)H, dehydrogenases (NQOs), and HO-1 [9, 10]. It has been well-known that phosphorylation of Nrf2 at tyrosine 568 is attributed to the nuclear export of Nrf2. Furthermore, up-regulation of p62 can bind to Keap1 thereby disrupting the interaction between Nrf2 and Keap1, leading to release of Nrf2 from Keap1, eventually, facilitating Nrf2 translocation from cytoplasm to nucleus [11, 12]. Collectively, Nrf2 signaling can not only manipulate redox signaling but also attenuate inflammation-associated pathogenesis in many diseased conditions including rheumatoid arthritis, asthma and atherosclerosis [13–15].

In the recent decades, increasing attention has been given to the study of Nrf2 signaling, especially in macrophages, for screening of active components from medicinal plants for anti-inflammation. For the first time, in 2011, it was reported that gedunin (GDN), an analog of 7-DGD, activated Nrf2 signaling based on an Neh2-luciferase reporter assay [16]. Also, GDN was found to be an inhibitor of heat shock protein 90 (Hsp90) and an activator of the transcription factor heat shock factor 1 (hsf1) [17, 18]. However, the anti-inflammatory effects of 7-DGD *in vivo* has not been studied till now. In the current work, we for the first time determined the anti-inflammatory effect and elucidated underlying mechanism of 7-DGD *in vivo* and *in vitro*.

## RESULTS

### 7-DGD mediates the anti-inflammatory effect on LPS-induced septic shock *in vivo* without significant cytotoxicity on macrophages

**Figure 1:**
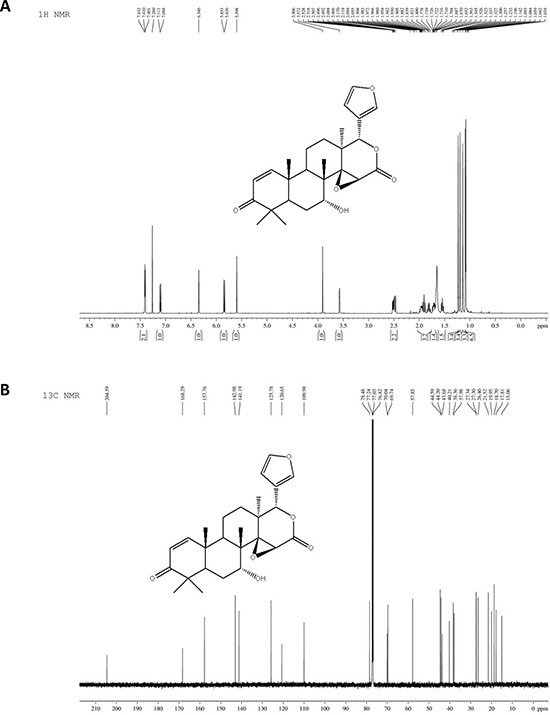
The structure of 7-deacetylgedunin (7-DGD) ^1^H NMR spectrum of 7-DGD (**A**). ^13^C NMR spectrum of 7-DGD (**B**).

**Figure 2:**
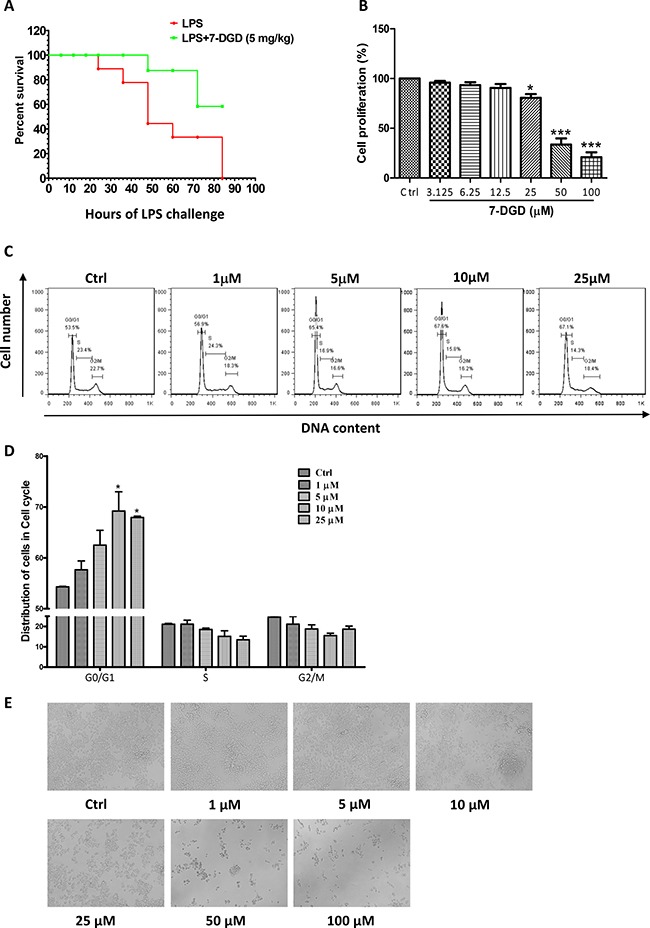
7-DGD alleviated the mortality of LPS-induced endotoxin shock in mice 8–11 week-old female C57BL/6 mice were injected intraperitoneally with LPS (20 mg/kg) in the presence of 7-DGD (5 mg/kg, *n* = 7) or vehicle (*n* = 8) (**A**). 7-DGD inhibited RAW 264.7 cells proliferation (**B** and **E**). 7-DGD induced RAW264.7 cell cycle arrest (**C**–**D**). Data were obtained from three independent experiments and the results were expressed as mean ± SEM. *p < 0.05, **p < 0.01, ***p < 0.001 vs control.

7-deacetylgedunin (7-DGD) (> 98% purity, verified by HPLC) was isolated from the fruits of Toona sinensis (A. Juss.) Roem. Its structure was elucidated on the basis of spectroscopic analysis (NMR data shown in Figure 1A and 1B). It was recently reported that gedunin, an analog of 7-DGD, has potential to inhibit the production of TNF-α, IL-6 and NO [19]. We therefore determined whether 7-DGD could ameliorate the pathogenesis of LPS-induced septic shock in mice, although the anti-inflammatory effect of the compound has not been investigated in animal. In the experiment, C57BL/6 mice were intraperitoneally injected with 5 mg/kg 7-DGD once per day for consecutive two days, followed with the treatment of LPS. As shown in Figure 2A, the results showed that all of the mice-treated by LPS were died within 84 h, and 40% mice administrated with 7-DGD were survived, showing that 7-DGD has anti-inflammatory function *in vivo*.

Activation of macrophages are heavily involved in the initiation and progression of inflammation [20]. Therefore, we further determined if the anti-inflammatory effect of 7-DGD resulted from the toxicity on macrophage. The results showed that 50 μM 7-DGD suppressed cell viability by 67% compared to the vehicle-treated cells (Figure 2B), and the dead cells were clearly observed under the microscope. When the cells were treated with 7-DGD at 100 μM, the suppression reached 80%, and the dead cells were more abundant (Figure 2E). However, treatment with 25 μM 7-DGD did not induce visible cell death, although the number of the viable cells was less than non-treated cells, indicating that 7-DGD probably inhibited macrophage proliferation rather than killed the cells. We further examined if the inhibitory effect of 7-DGD on macrophage proliferation attributed to the cell cycle arrest. It was found that 7-DGD at 10 μM and 25 μM concentrations significantly induced the cell cycle arrest of RAW264.9 cells at G0/G1 phase (Figure 2C–2D), suggesting that anti-inflammatory effect of 7-DGD may result from inhibition on the production of inflammatory mediators rather than cytotoxicity.

### 7-DGD significantly inhibits mRNA and protein expression of iNOS, and mRNA expression of IL-1β and TNF-α in macrophages

**Figure 3:**
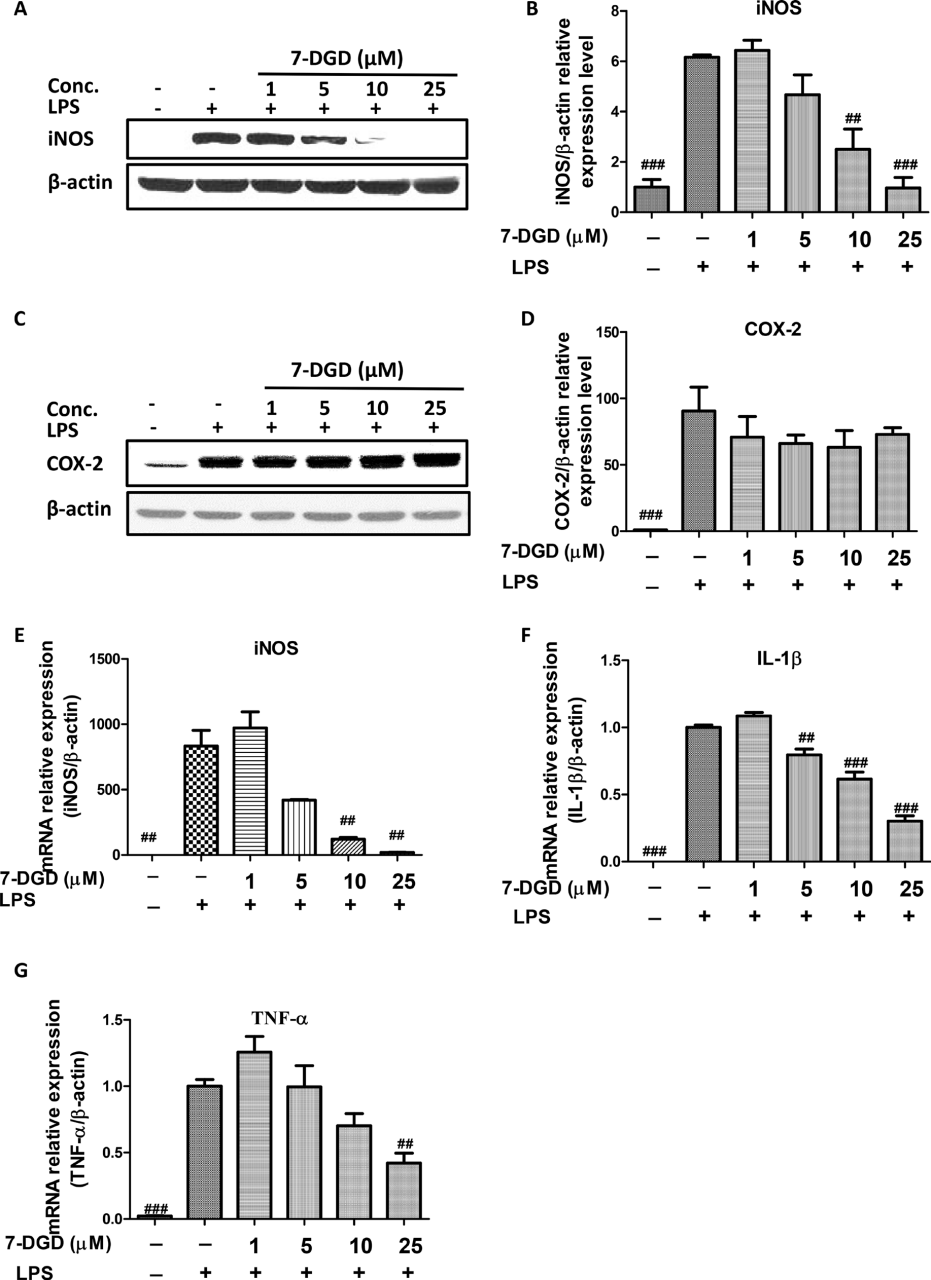
7-DGD inhibited mRNA and protein expression of iNOS, and mRNA expression of IL-1β and TNF-α in RAW 264.7 cells induced by LPS The effect of 7-DGD on iNOS protein expression (**A**–**B**). The effect of 7-DGD on COX-2 protein expression (**C**–**D**). The effect of 7-DGD on iNOS mRNA expression (**E**). The effect of 7-DGD on mRNA expression of IL-1β and TNF-α (**F**–**G**). Data were obtained from three independent experiments and the results were expressed as mean ± SEM, ^#^*p* < 0.05, ^##^*p* < 0.01, ^###^*p* < 0.001 *vs* LPS group.

iNOS and COX-2 play pivotal roles in oxidative and inflammatory events [20]. We examined whether 7-DGD could influence iNOS and COX-2 expression. The results showed that 7-DGD could almost completely attenuate iNOS protein expression at 10 μM (Figure 3A–3B), while COX-2 protein expression remained unchanged up to 25 μM concentration (Figure 3C–3D). Therefore, we further determined whether the inhibitory effect of 7-DGD on iNOS expression resulted from transcriptional suppression. In coincided with the results, 7-DGD significantly inhibited the mRNA expression level of iNOS (Figure 3E). It is well known that the cytokines, including TNF-α and IL-1β, play a significant role in inflammatory activities. Accordingly, the effects of 7-DGD on the mRNA expression of TNF-α and IL-1β were determined, and the results demonstrated that 7-DGD could significantly inhibit IL-1β and TNF-α mRNA expression (Figure 3F–3G), suggesting that 7-DGD has anti-inflammatory potential in macrophages.

### 7-DGD enhances NQO1, HO-1 and UGT1A1 mRNA expression, and HO-1 protein expression level with accumulation of Nrf2 in macrophages

**Figure 4:**
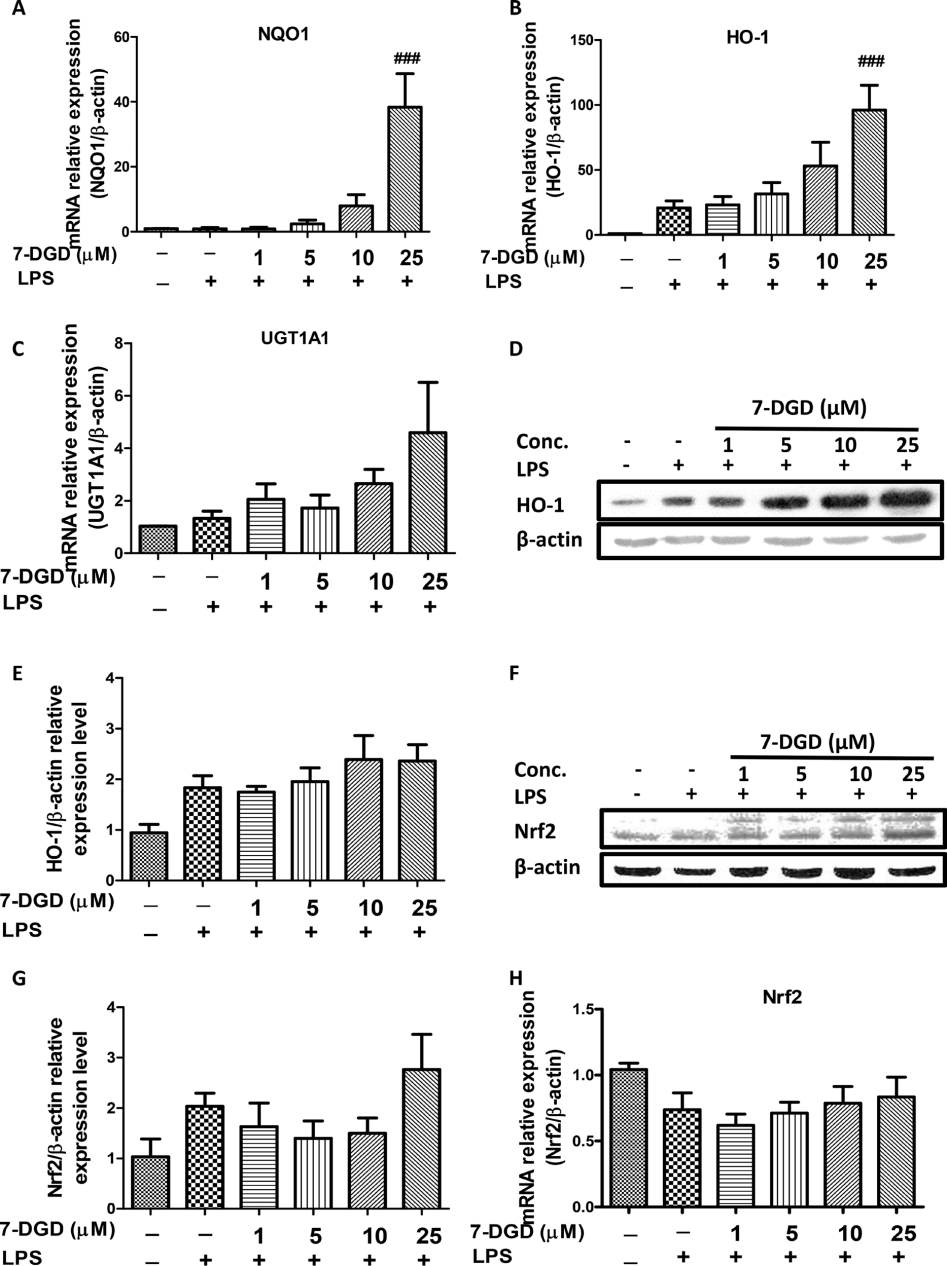
7-DGD up-regulated anti-oxidant enzymes mRNA and protein expression, as well as Nrf2 accumulation in RAW264.7 cells induced by LPS The effect of 7-DGD on NQO1, HO-1 and UGT1A1 mRNA expression (**A**–**C**). The effect of 7-DGD on HO-1 protein expression (**D**–**E**). The effect of 7-DGD on Nrf-2 protein expression (**F**–**G**). The effect of 7-DGD on Nrf-2 mRNA expression (**H**). Data were obtained from three independent experiments and the results were expressed as mean ± SEM. ^#^*p* < 0.05, ^##^*p* < 0.01, ^###^*p* < 0.001 *vs* LPS group.

Decreased HO-1 and other anti-oxidant enzymes can induce over-production of iNOS and COX-2, and up-regulation of HO-1 has been reported to exert anti-inflammation as well as anti-oxidant effects [21]. Therefore, we further examined whether or not the inhibitory effect of 7-DGD is resulted from up-regulation of NQO1, HO-1 and UGT1A1. As anticipated, the results showed mRNA expression of NQO1, HO-1, and UGT1A1 was increased by 7-DGD, especially at 25 μM (Figure 4A–4C). We further verified the effect of 7-DGD on HO-1 protein expression in RAW264.7 with LPS stimulation, and the results revealed that 7-DGD could strongly facilitate HO-1 expression in a dose-dependent manner in RAW 264.7 with LPS stimulation (Figure 4D–4E), indicating that suppression of macrophage activation induced by 7-DGD relies on the up-regulation of the anti-oxidative enzymes expression. It was reported that the anti-oxidative enzymes including NQO1, HO-1 and UGT1A1 are regulated by Nrf2 [13, 15]. Interestingly, we found that 7-DGD induced accumulation of Nrf2 in the cells (Figure 4F–4G) without affecting mRNA expression level of Nrf2 (Figure 4H), indicating that 7-DGD probably prevented Nrf2 ubiquitination rather than overexpression.

### 7-DGD promotes Nrf2 localization in the nucleus of macrophages

**Figure 5:**
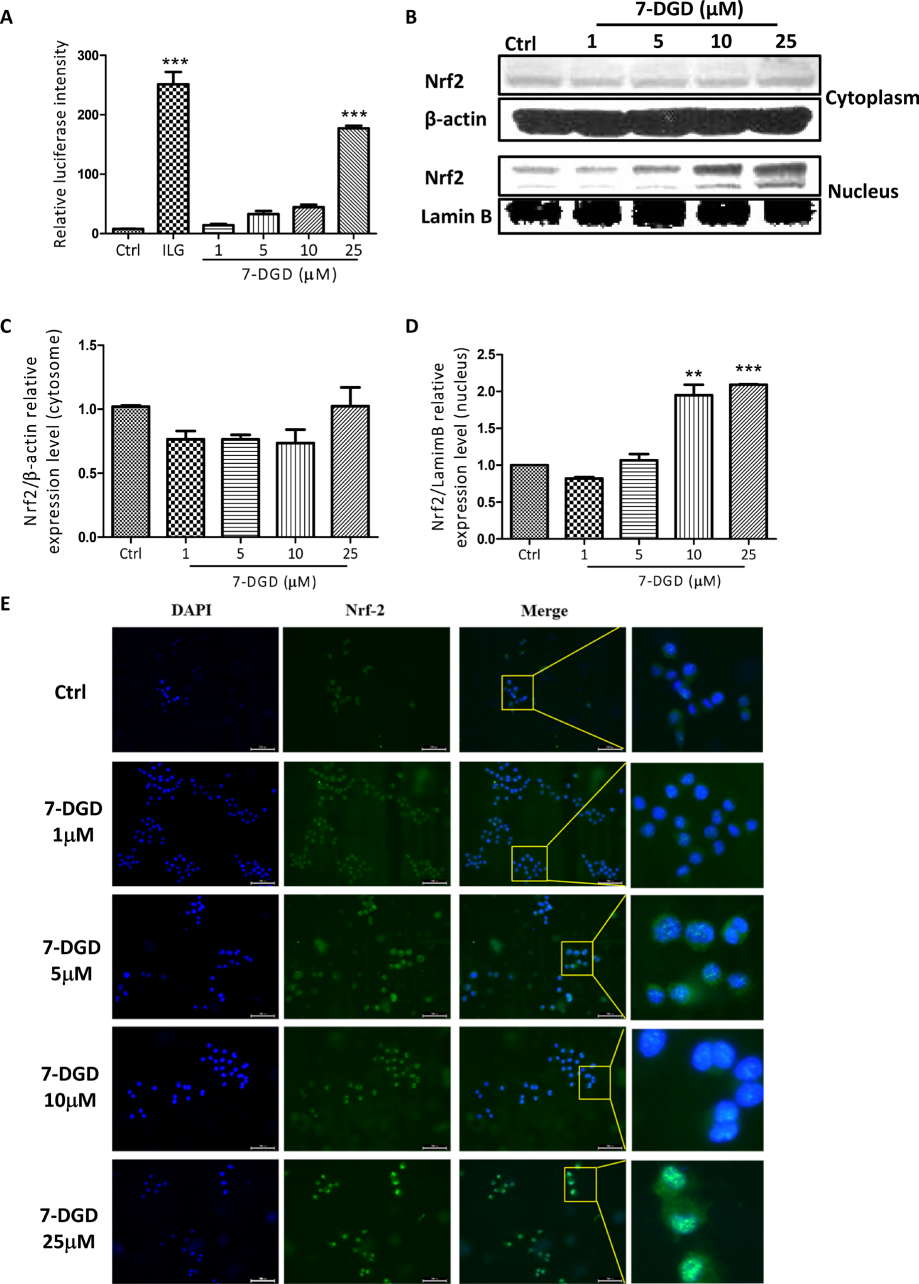
7-DGD facilitated Nrf2 nucleus translocation in RAW264.7 cells The effect of 7-DGD on ARE luciferase activity (**A**). The effect of 7-DGD on Nrf2 nucleus translocation determined by western blotting (**B**–**D**) and immunofluorescence (**E**) in RAW264.7 cells. Data were obtained from two or three independent experiments and the results were expressed as mean ± SEM. *p < 0.05, **p < 0.01, ***p < 0.001 *vs* control.

As 7-DGD could obviously facilitate Nrf2 accumulation in cytoplasm, we further determined whether the compound could promote Nrf2 nucleus translocation. Additionally, it is well known that Nrf2 dimerizes with small Maf proteins and binds to ARE to activate these anti-oxidant enzymes transcription when it translocates to cell nucleus. Before determining the effect of 7-DGD on Nrf2 nucleus translocation, we examined the effect of 7-DGD on ARE luciferase activity on HepG2-C8 cells, the stably transfected cells with an ARE-luciferase reporter vector. As shown in Figure 5A, the reference compound, isoliquiritigenin (ILG), could significantly increase ARE luciferase activity, which is coincided with previous study [22]. As expectation, 7-DGD up-regulated the luciferase activity of ARE (Figure 5A). Furthermore, it was reported that the function of the activated Nrf2 relies on its translocation from the cytoplasma into the nucleus [9, 15], indicating that 7-DGD probably has the ability to promote Nrf2 nucleus translocation. So, it is valuable to examine the effect of 7-DGD on Nrf2 translocation into the nucleus of macrophages by western blotting and immunocytochemistry assays. As shown in Figure 5B–5D, 7-DGD could obviously increase Nrf2 translocation from cytoplasm to the nucleus of RAW264.7 cells. In coincided with the results, the immunocytochemistry analysis showed that 7-DGD intensively induced the Nrf2 localization in the nucleus of 7-DGD-treated cells at 10 μM and 25 μM (Figure 5E). These findings suggested that 7-DGD can promote Nrf2 localization in the nucleus of macrophages and in turn up-regulate anti-oxidative gene transcription to inhibit inflammation.

### 7-DGD activates Nrf2 signaling by regulation of Keap1 and p62 expression

**Figure 6:**
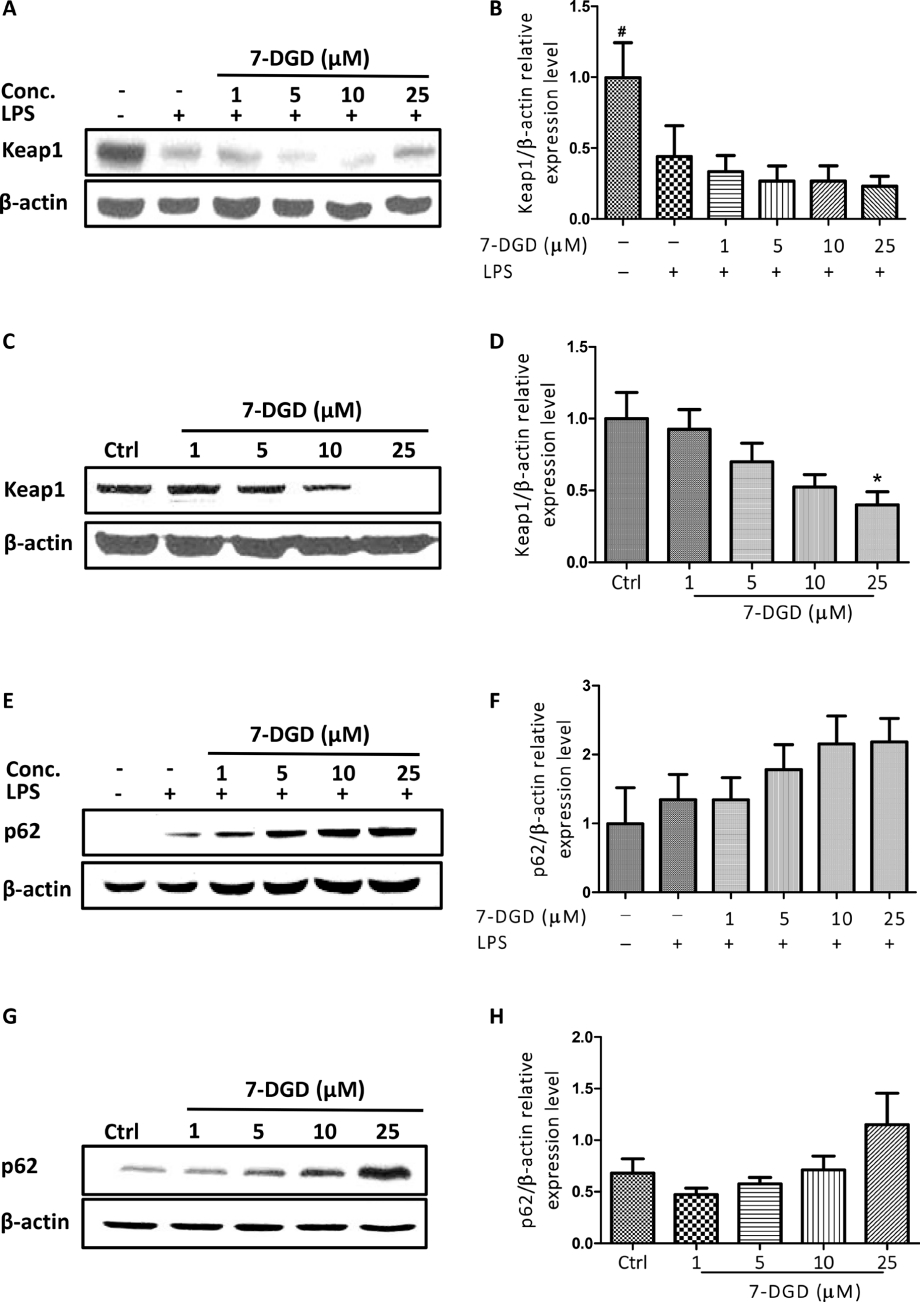
7-DGD induced Keap1 degradation and promoted p62 expression The effect of 7-DGD on Keap-1 expression in RAW264.7 cells with (**A**–**B**) or without (**C**–**D**) LPS stimulation. The effect of 7-DGD on p62 expression in RAW264.7 cells with (**E**–**F**) or without (**G**–**H**) LPS stimulation. Data were obtained from three independent experiments and the results were expressed as mean ± SEM. *p < 0.05, **p < 0.01, ***p < 0.001 *vs* control, ^#^*p* < 0.05, ^##^*p* < 0.01, ^###^*p* < 0.001 *vs* LPS group.

Nrf2 is normally sequestered in the cytoplasm by a protein known as Keap1. When Keap1 is challenged, blockage of Nrf2 ubiquitylation is induced and Nrf2 translocation from the cytoplasm to the nucleus is increased. Considering the crucial role of Keap1 in Nrf2, the effect of 7-DGD on Keap1 expression was determined. As shown in Figure 6A–6D, 7-DGD could intensively inhibit Keap1 expression in RAW264.7 cells with or without LPS stimulation, indicating that 7-DGD induced Nrf2 accumulation probably through suppressing Keap1 expression.

Recently, p62 was reported to activate Nrf2 by inactivation of Keap1 through interaction between the Nrf2-binding site on Keap1 at the Keap1-DC domain and p62-KIR [12]. We further examined the effect of 7-DGD on p62 expression and found that 7-DGD significantly promoted p62 expression in RAW264.7 macrophages with or without LPS stimulation in a dose-dependent manner (Figure 6E–6H), suggesting that 7-DGD facilitated Nrf2 accumulation and nucleus translocation due to up-regulation of p62 and inhibition of Keap1.

## DISCUSSION AND CONCLUSIONS

In the recent decades, an increasing number of reports have indicated that inflammatory responses are a common pathogenesis of a variety of diseases [23], in which macrophages function as critical participants in inflammatory and autoimmune processes. A number of pro-inflammatory proteins and enzymes, such as iNOS, COX-2 and Keap1/Nrf2/HO-1 signaling, in macrophages have been found to influence and attenuate inflammation-associated pathogenesis of a variety of diseases [14].

The anti-inflammatory effect of 7-DGD, a limonoid isolated from *T. sinensis*, has not been investigated yet. In the current study, we for the first time demonstrated that 7-DGD alleviated mice mortality induced by LPS, indicating that the compound has anti-inflammatory potential *in vivo*. The macrophage cell line, RAW 264.7, was employed to explore the underlying mechanism of 7-DGD. The results of proliferation and cell cycle assay revealed that the anti-inflammatory effect of the compound cannot be attributed to cytotoxicity in macrophages.

It is well-known that iNOS is one of the key inflammatory mediators associated with pathogenesis and pathological changes in many diseased conditions [24, 25], In the current work, we found that 7-DGD possesses the promising function of inhibiting iNOS expression in RAW264.7 macrophages stimulated by LPS for 24 h, while it has no influence on COX-2 expression, indicating that 7-DGD has selective inhibitory effect on iNOS expression. In addition, the inhibitory effect of 7-DGD on TNF-α and IL-1β showed that the compound has the anti-inflammatory effect in macrophages.

Although the effect of 7-DGD on Nrf2 signaling has not been studied, GDN, the 7-DGD analog, has been served as an Nrf2 activator in the Neh2-luciferase reporter assay [16]. It was reported that disruption or loss of Nrf2 signaling causes enhanced susceptibility not only to oxidative and electrophilic stresses but also to inflammatory tissue injuries [26]. Furthermore, Nrf2 knockout mice exhibited significantly increased iNOS expression compared with their wild-type counterparts [27, 28], implying that activation of Nrf2 probably contributed the anti-inflammatory effect of 7-DGD. Therefore, we conducted intensive studies on 7-DGD with respect to the correlation between iNOS expression and Keap1/Nrf2/HO-1 signaling. When Nrf2 is activated, it binds to ARE located in the promoter region of genes encoding HO-1, NOQ1 and UGT1A1. Our results clearly demonstrated that 7-DGD significantly enhanced HO-1, NOQ1 and UGT1A1 mRNA expression. Among these enzymes regulated by Nrf2, HO-1 has pronounced anti-oxidative and anti-inflammatory properties, and it is a crucial modulator to regulate the innate immunity and inflammation [29]. In the study, we found 7-DGD obviously up-regulated HO-1 protein expression level.

Under the basal resting condition, Nrf2 is sequestered in the cytoplasm by Keap1, which functions as a negative modulator of Nrf2 by promoting ubiquitination and proteasomal degradation of Nrf2. In the study, 7-DGD significantly inhibited Keap1 expression and enhanced Nrf2 expression in cytoplasm. Interestingly, 7-DGD has not obvious effect on Nrf2 mRNA expression level, suggesting that the compound cannot affect Nrf2 transcription. Therefore, Nrf2 accumulation in cytoplasm induced by 7-DGD was attributed to inhibition on Keap1.

It has been well-known that p62 functions as a selective autophagy receptor for degradation of ubiquitinated substrates. It was reported that mTORC phosphorylates S349 of p62 and increases binding affinity of p62 to Keap1, resulting in the escape of Nrf2 from the Keap1 interaction. Free Nrf2 enables activation of various target genes, including p62, which creates a positive feedback loop in the p62-Keap1-Nrf2 axis [12, 30]. In our current study, 7-DGD increased Nrf2 nucleus localization and in turn up-regulated p62 mRNA and protein expression. On the other hand, TRIM21, an ubiquitin E3 ligase negatively regulates the p62-Keap1-Nrf2 axis by undoing p62 oligomerization. According to the literature reports and our results, we speculated that 7-DGD probably targets on mTOR complex 1 (mTORC1) or TRIM2, and then activates p62-Keap1-Nrf2 axis to mediate anti-inflammatory effect.

Taken together, for the first time, we reported the anti-inflammatory effect of 7-DGD *in vivo* and *in vitro*. Mechanistic study explored that 7-DGD suppressed the macrophages proliferation through arresting cell cycle at the G0/G1 phase, and inhibited iNOS expression *via* activation Nrf2/ARE/HO-1 signaling. The effect of 7-DGD on Nrf2 accumulation and nucleus translocation resulted from inhibition of Keap-1 expression and up-regulation of p62 expression, and then enhanced HO-1, NOQ1 and UGT1A1 mRNA expression and HO-1 protein expression level, finally mediated anti-inflammatory effect. In conclusion, 7-DGD suppressed inflammation *in vivo* and *in vitro* via activation of Keap1/Nrf2/HO-1 signaling. In future, 7-DGD is valuable for further investigation as an anti-inflammatory agent.

## MATERIALS AND METHODS

### Cells and reagents

RAW264.7 cells were purchased from American Type Culture Collection (ATCC, Manassas, VA, USA) and were cultured in DMEM (Gibco, Paisley, UK) with 10% (v/v) fetal bovine serum (FBS; Gibco, Paisley, UK), penicillin (100 units/ml) and streptomycin (100 μg/ml). Sodium dodecyl sulfate (SDS) and chemiluminescence (ECL) were brought from Life Technologies (Carlsbad, California, USA). Paraformaldehyde (PFA) and lipopolysaccharide (LPS) was provided by Sigma (St. Louis, MO, USA). The HepG2-C8 cell line was kindly provided by Prof. Ah-Ng Tony Kong, Rutgers University, USA. Both RAW264.7 cells and HepG2-C8 cells were cultured at 37°C in a 5% CO_2_ atmosphere. Primary antibodies against iNOS, COX-2, HO-1, Nrf2, Keap1 and p62 were obtained from Cell Signaling Technologies (Danvers, MA, USA), and the antibody against β-actin was obtained from Santa Cruz Technology (Dallas, Texas, USA). Luciferase assay kits were purchased from Promega (Madison, Wisconsin, USA). Lipopolysaccharide (LPS) was provided by Sigma (St. Louis, MO, USA). 7-DGD was dissolved in DMSO at a stock concentration of 100 mM and stored at −40°C.

### LPS-induced septic shock model

C57BL/6 mice were obtained from the Chinese University of Hong Kong, and were fed with a standard diet and libitum. Housing conditions and all *in vivo* experiments were approved under the regulation of the Laboratory Animal Research Committee Guidelines of Macau University of Science and Technology. For the induction of septic shock, 8–11 week-old C57BL/6 mice were randomly divided to 7-DGD or vehicle treatment groups. Mice were pretreated with or without 5 mg/kg 7-DGD at 24 h and 1 h before the mice were intraperitoneally injected with 20 mg/kg LPS. The survival of mice was recorded in the following 84 h.

### Cytotoxicity assay

RAW264.7 cells were seeded into 96-well plates for overnight and treated with 7-DGD at concentrations of 0, 3.125, 6.25, 12.5, 25, 50, and 100 μM for 24 h before reacting with MTT (5 mg/mL) for 4 h. The solvent (10% SDS, 50% N, N-dimethyl formamide) was added to the mixed solution in the 96-well plates to dissolve the purple precipitate. Cell viability was analyzed in each well at 570 nm using an OD plate reader (Infinite M200 PRO, Germany) after overnight incubation. The formula was used to calculate cell viability according to the formula, cell viability (%) = OA _treated_/OA _control_.

### Flow cytometric analysis

To determine the influence of 7-DGD on the cell cycle of RAW264.7 macrophages, RAW264.7 cells (1 × 10^6^/well) were treated with 7-DGD at concentrations of 1, 5, 10, and 25 μM for 24 h, and the cells were then collected and washed 3 times with cold PBS and fixed with 70% ethanol for 1 h. Subsequently, the total DNA contents were stained with PBS containing propidium iodide and 100 μg/ml RNase I for 30 min at 37°C. Finally, the cell cycle was analyzed using a FACScan flow cytometer (Becton Dickinson, San Jose, CA, USA).

### Luciferase reporter activity assay

To examine the effect of 7-DGD on ARE, ARE luciferase assay was performed in HepG2-C8 cells which were stably transfected the ARE-luciferase vector. HepG2-C8 cells (5 × 10^4^/well) were seeded in 24-well plates for 24 h and then co-incubated with indicated concentrations of 7-DGD or isoliquiritigenin (ILG) for another 24 h. The activity of ARE luciferase was analyzed using the luciferase assay kit. Briefly, HepG2-C8 cells were collected and lysed by the reporter lysis buffer (50 μl/well) and centrifuged at 12,000 *g* for 10 min, and 25 μl cell lysate supernatant was used for the determination of the luciferase activity using a Sirius luminometer (Bertold Detection System GmbH, Pforzheim, Germany). The remaining cell lysates were used for BCA protein quantification. The luciferase activity was normalized by the BCA protein quantification assay. The formula used to calculate the luciferase activity is according to the following formula, relative luciferase activity = (luciferase activity/protein concentrations)_treated_ / (luciferase activity/protein concentrations)_control_. The experiments were obtained from three independent experiments, and the results are presented as the mean ± SEM.

### Protein extraction and western blotting

To examine the influence of 7-DGD on iNOS and COX-2 expression, RAW264.7 cells (1 × 10^6^/well) were pretreated with or without 7-DGD (1, 5, 10, 25 μM) for 1 h and then co-incubated with 100 ng/ml LPS for another 24 h. To determine the effect of 7-DGD on HO-1, Keap1, Nrf2 and p62 expression, the cells were pretreated with or without 7-DGD for 1 h and then stimulated with 100 ng/ml LPS for 24 h. After the cells were collected and lysed by 1 × RIPA containing 1× protease inhibitor (Roche, USA), and the total protein concentrations were quantified using a Bio-Rad assay. To determine the effect of 7-DGD on Nrf2 nucleus translocation, the cells were incubated with 7-DGD for 24 h, and then the cytoplasmic and nucleus extracts were prepared using NE-PER nuclear cytoplasmic extraction kit (Thermo, Pierce, USA).

After preparation, the extracts were subjected to 10% SDS-PAGE gel and were separated at 120 V for 90 min before transferring to nitrocellulose (NC) membranes for another 2 h. Then, the NC membranes containing protein were blocked with 5% fat free milk in Tris-buffered saline with 0.1% Tween 20 (TBST). Subsequently, the membranes were incubated with primary antibodies overnight followed by incubation with HRP-conjugated secondary antibodies for 1 hour. A chemiluminescence (ECL) detection system was used to determine the antibody-bound proteins on the membrane.

### Quantitative real-time polymerase chain reaction

To determine the levels of iNOS, IL-1β, TNF-α, HO-1, Nrf2, NQO1 and UGTIA1 mRNA expression, RAW264.7 cells (1 × 10^6^/well) were plated in 6-well plates and pre-incubated with 7-DGD for 1 h, followed by stimulation with LPS (100 ng/ml) for another 24 h. Then, the total RNA of the cells was collected in 1 ml/well Trizol (Invitrogen, Grand Island, NY). The concentration of total RNA was calculated by UV spectrophotometry at 260/280 nm (Nanodrop, Thermo). The expression levels of iNOS, IL-1β, TNF-α, HO-1, Nrf2, NQO1 and UGTIA1 mRNA were quantified by quantitative real-time polymerase chain reaction (qPCR) according to previous description.

The mouse primer sequences were listed as following:

**Table:** 

Gene	Primer sequence
iNOS	forward: 5′-GTGGTGACAAGCACATTT GG-3′ reverse: 5′-GGCTGGACTTTTCACTCT GC-3′
HO-1	forward: 5′-CCCACCAAGTTCAAACAGC TC-3′ reverse: 5′-AGGAAGGCGGTCTTAGCC TC-3′
Nrf2	forward: 5′-AGC AGG ACA TGG AGC AAG TT-3′ reverse: 5′-TTC TTT TTC CAG CGA GGA GA-3′
NQO1	forward: 5′-TTCTGTGGCTTCCAGGTCT T-3′ reverse: 5′-AGGCTGCTTGGAGCAAAAT A-3′
UGT1A1	forward: 5′-GTGGCCCAGTACCTGACT GT-3′ reverse: 5′-CGATGGTCTAGTTCCGGTG T-3′
TNF-α:	forward: 5'-TATGGCTCAGGGTCCAAC TC-3′ reverse: 5'-CTCCCTTTGCAGAACTCAG G-3′
IL-1β:	forward: 5'-TTGACGGACCCCAAAAGAT G-3′ reverse: 5'-AGAAGGTGCTCATGTCCT CA-3′
β-actin	forward: 5′-TGCTCGAGATGTCATGAAG G-3′ reverse: 5′-TTGCGCTCATCGTAGGCTT T-3′

### Immunocytochemistry

To analyze the effect of 7-DGD on Nrf2 translocation into the nucleus, we conducted the immunocytochemistry assay. Briefly, RAW264.7 cells (1 × 10^5^/well) were seeded on coverslips in 6-well plates and incubated overnight. The cells were then treated with different concentrations of 7-DGD for 24 h. Cells were washed 3 times with cold PBS and fixed with 4% PFA for 15 min. After washed 3 times with cold PBS and permeabilized with 0.1% Triton X-100 for 5 min, the cells were stained with DAPI for 30 min. Then, the cells were incubated with Nrf2 antibody overnight at 4°C followed by incubation with secondary antibody for 2 h at room temperature. After the coverslips were dried, the cells were mounted onto glass slides and analyzed by fluorescent microscopy.

### Statistical analyses

The data are presented as the means ± SEM. ANOVA with a Tukey's post-hoc test in GraphPad Prism was used to analyze the significance of the differences; *p < 0.05, **p < 0.01, ***p < 0.001, ^#^*p* < 0.05, ^##^*p* < 0.01, and ^###^*p* < 0.001 were considered statistically significant.
